# Range Analysis of Thermal Stress and Optimal Design for Tungsten-Rhenium Thin Film Thermocouples Based on Ceramic Substrates

**DOI:** 10.3390/s17040857

**Published:** 2017-04-14

**Authors:** Zhongkai Zhang, Bian Tian, Qiuyue Yu, Peng Shi, Qijing Lin, Na Zhao, Weixuan Jing, Zhuangde Jiang

**Affiliations:** State Key Laboratory for Mechanical Manufacturing Systems Engineering, Xi’an Jiaotong University, Xi’an 710049, China; z.zhongkai@stu.xjtu.edu.cn (Z.Z.); yuqiuyue@stu.xjtu.edu.cn (Q.Y.); spxjy@mail.xjtu.edu.cn (P.S.); mimilqj@163.com (Q.L.); zn2015@stu.xjtu.edu.cn (N.Z.); wxjing@mail.xjtu.edu.cn (W.J.); zdjiang@mail.xjtu.edu.cn (Z.J.)

**Keywords:** TFTCs, range analysis, optimal design

## Abstract

A thermal stress range analysis of tungsten-rhenium thin film thermocouples based on ceramic substrates is presented to analyze the falling off and breakage problems caused by the mismatch of the thermal stresses in thin film thermocouples (TFTCs) and substrate, and nano-indentation experiments are done to measure and calculate the film stress to compare with the simulation results. Optimal design and fabrication of tungsten-rhenium TFTCs based on ceramic substrates is reported. Static high temperature tests are carried out, which show the optimization design can effectively reduce the damage caused by the thermal stress mismatch.

## 1. Introduction

The temperature parameters of aero-engines are a main influencing factors of the performance and safety of aeroplanes in the aero-engine development process. In recent years, the working temperature of hot components of new aero-engines has grown to achieve a higher thrust weight ratio, for example, the temperature before the turbine of a F119 is up to 1950 K. To reliably prevent over-temperature damage, controlling the temperature in the combustors and turbine blades of aero-engines is therefore an important requirement. Although the traditional test methods can be used in testing these high temperatures, this is still not suited for the harsh environment in aero-engines. Sapphire fiber temperature sensors are hard to apply in online monitoring. Infrared radiation devices are complex and their precision is not enough for use in online measurement of hot components in harsh environments such as aero-engines. Furthermore, the armored thermocouple insulation layer decreases, easily leading to leakage currents, resulting in shunting errors and drift control problems [1]. In addition, the larger size limits the application of armored thermocouple. With the development of micro-electro mechanical system (MEMS) technology, thin film thermocouples (TFTCs) have been developed on different materials and applied in the aerospace field. Compared with traditional thermocouples, TFTCs have typical two-dimensional characteristics, which offers advantages of small thermal capacity and rapid response to measure transient temperature changes accurately [2]. The thickness of the hot junction is micro- or nano-scale, which makes it more suitable for the temperature measurement of hot components in aero-engines. Besides, film thermocouples can be directly sputter-deposited onto the surface and have fairly thin thicknesses of approximately a few micrometers. Therefore, TFTCs provide minimal interference with the gas flow over the surface and do not require that the installation surface be structurally changed.

At present, the material system of film thermocouples has been diversified with the increase of the working temperature. Tungsten-rhenium alloy has a high melting point of 2700 K and appropriate thermocouple characteristics, which can be used in TFTCs for detecting high temperatures. We have presented a new tungsten-rhenium TFTC structure based on MEMS technology [3]. However, there’s a big difference in the thermal deformation of the film and substrate because the thermal stress of the film changes at high temperature after combining with the substrate [4]. Therefore, the mismatch of the thermal stress in TFTCs and substrate leads to falling off and breakage problems. The relationship between substrate materials and dimension parameters is analyzed to optimally design tungsten-rhenium TFTCs in this paper. Firstly, different ceramic substrates and film sizes have been chosen to do a thermal stress range analysis, then the relationship between substrate material, and TFTC dimensional parameters is analyzed to provide an optimal design. TFTCs are prepared for a comparison test. Lastly, static high temperature tests are carried out to validate the optimal design results.

## 2. Sensor Design and Modeling

### 2.1. Schematic and Structure Design

**Figure 1:**
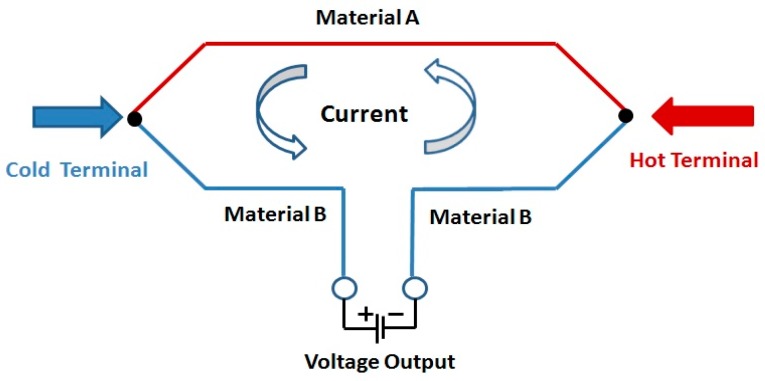
Schematic diagram of thermocouples.

TFTCs are usually made by two kinds of material, which are connected into a closed-loop. When the hot terminal is affected by temperature, and once there exists a temperature gradient field between the hot and cold terminals, the voltage could be measured from the cold terminal. The schematic diagram of these thermocouples is shown in Figure 1. Tungsten-rhenium alloys of different compositions were used such as tungsten-3% rhenium (W-3Re) type and tungsten-25% rhenium (W-25Re) type materials for the TFTC sensors.

**Figure 2:**
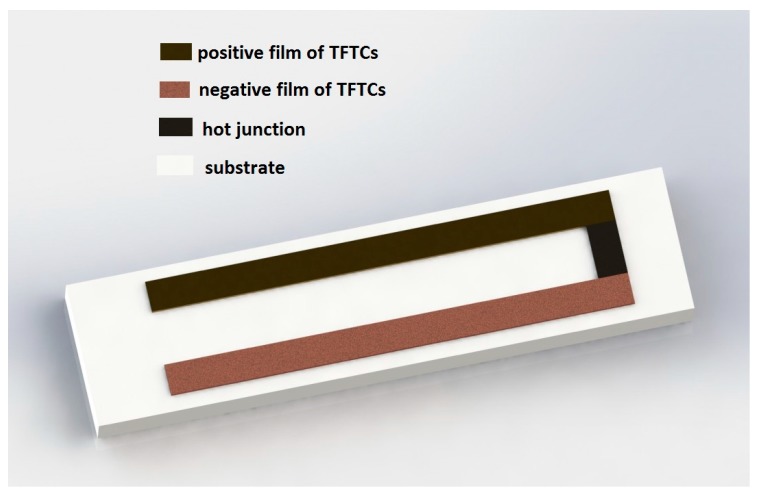
TFTCs sensor design.

As shown in Figure 2, the TFTC sensors are designed on a ceramic substrate. The positive film and negative film thermocouples made of tungsten-rhenium thin films are laid on the ceramic substrate. The positive film is made of tungsten-3% rhenium (W-3Re), and the negative film is made of tungsten-25% rhenium (W-25Re). The hot junction is made of W-3Re film and W-25Re film. Voltage is output from the end of the positive film and negative film.

### 2.2. Numerical and Simulation Model

Considering the substrate is a ceramic material which has a great strength, the thermal stress is not the main factor in the failure of the substrate. The stress finite element model of a TFTC sensor is set up to analyze the thermal stress of the tungsten-rhenium films on the substrate [5]. Due to the axial-symmetries of the problems describe here, the model is simplified into 2D. L, d_1_, d_2_ are the dimension parameters. L represents the length the film; d_1_ means the thickness of the film, and d_2_ means the thickness of the substrate. In the thermal stress finite element analysis (FEA), the isotropic, thermoplastic and orthotropic behavior of the material was considered in the tungsten-rhenium film combination. The simulations of thermal stresses were performed using finite element analysis. The material parameters in the model are listed in Table 1.

An analytical model, which shows the average thermal stress, is also set up for contrast. It’s in conjunction with Stoney’s equation for tension in metallic films, which would result in the following equation for thermal stress in thin film as [7]:

(1) $$\sigma_{th}=\frac{E_{ef}\int_{T_{r}}^{T_{D}}\left(\alpha_{s}-\alpha_{f}\right)dT}{1+4(E_{ef}/E_{es})(h/H)}$$

The definitions of the symbols in Equation (1) are given in Table 2.

## 3. Simulation and Optimization

**Figure 3:**
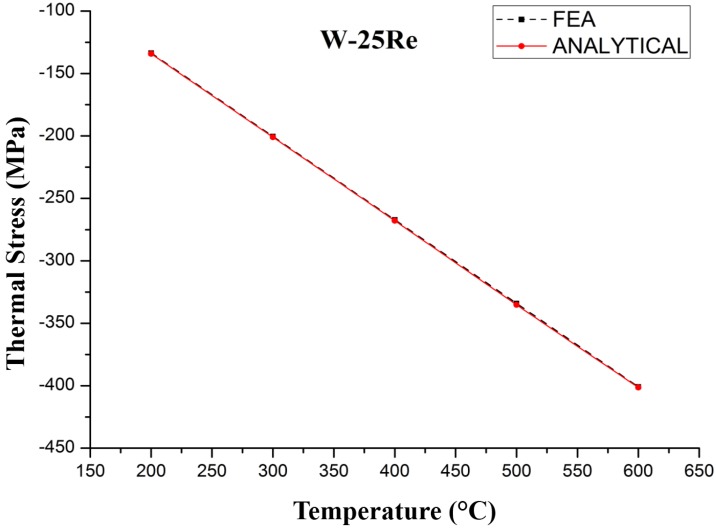
Thermal stress variation with temperature in W-25Re.

**Figure 4:**
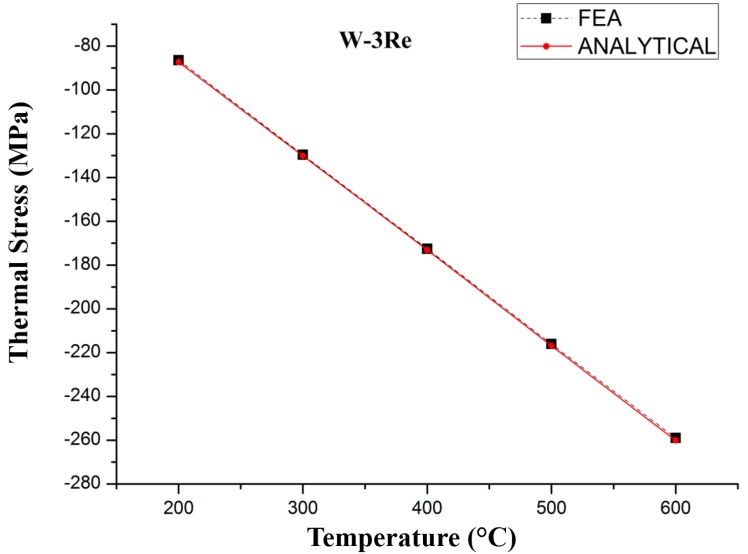
Thermal stress variation with temperature in W-3Re.

The relationship between the average thermal stress generated in TFTCs deposited on silicon carbide ceramic substrates and temperature is shown in Figure 3 and Figure 4. The thickness of tungsten-rhenium film (d_1_) is 1 μm, the thickness of the substrate (d_2_) is 100 μm, the length of the film and substrate is 200 μm. The physical parameters are chosen from Table 1. The error rate is less than 1%. The thermal stress varies with temperature, and the values calculated by FEA analysis are in accordance with the analytical model.

**Figure 5:**
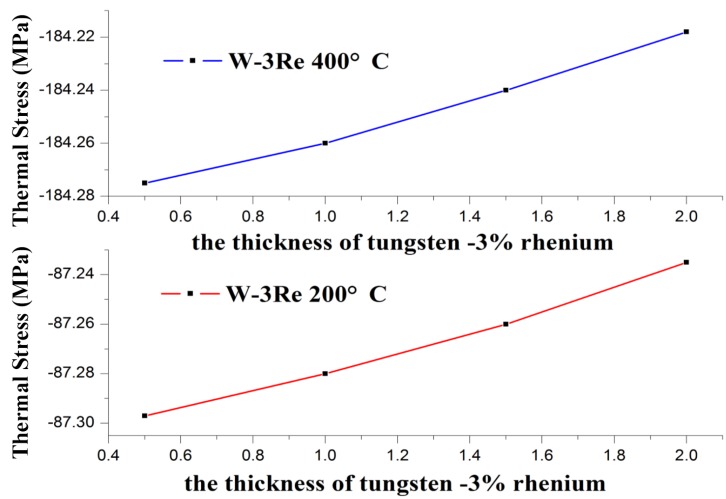
Thermal stress variation with temperature and the thickness of W-3Re film.

**Figure 6:**
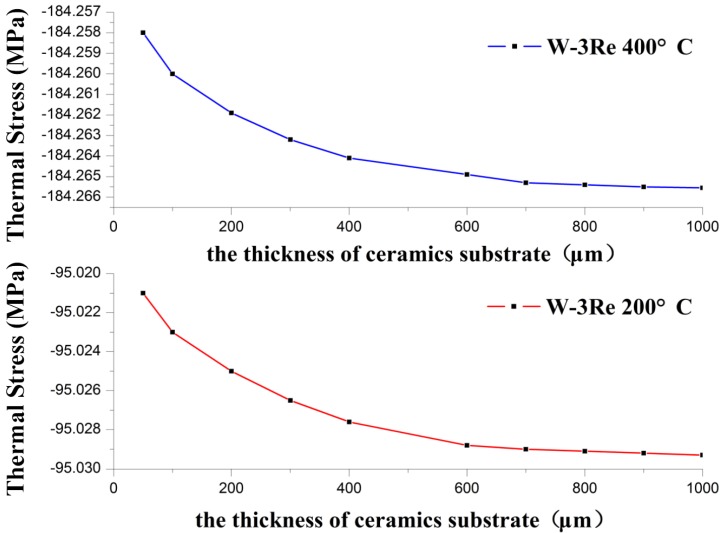
Thermal stress variation with temperature and the thickness of the substrate.

Thermal stress variation with the increasing thickness of W-3Re film is shown in Figure 5, and the variation with the increasing thickness of the substrate is shown in Figure 6, respectively. The material of the substrate is silicon carbide ceramic.

In Figure 5, the thickness of W-3Re (d_1_) ranges and d_2_ = 100 μm. In Figure 6, the thickness of the substrate (d_2_) ranges and d_1_ = 3 μm. It is observed that thermal stress increases with increased temperature. The reason is the coefficient of thermal expansion mismatch between film and substrate. The thickness of W-3Re film also influences the thermal stress of W-3Re film deposited on a substance, and with the increase of the thickness of W-3Re film, the thermal stress decreases [8]. This is due to the bending strain induced at higher thickness causing stress relaxation. In addition, thermal stress variation with temperature and the thickness of the substrate and W-3Re film has a smaller amplitude because the thickness of W-3Re film changes in a very small interval and the thickness of the substrate is much bigger than that of the W-3Re film, which was decided according to the application requirements.

**Figure 7:**
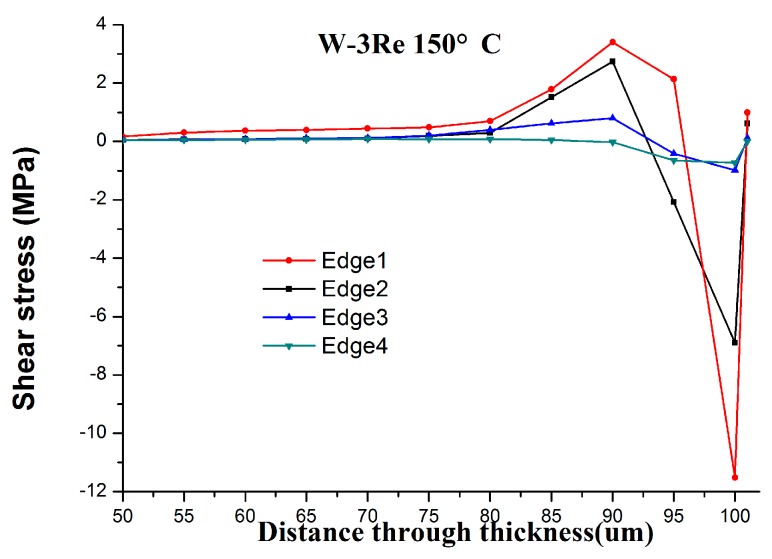
The distribution of shear stress (σ_xz_) of W-3Re with the distance through thickness at different position.

To analyze the distribution of shear stress(σ_xz_) of W-3Re with the distance through thickness at different position, we choose silicon carbide ceramic as the substrate and W-3Re as the film, d_1_ = 1 μm, d_2_ = 100 μm, the lower surface of substrate as base level, and lateral boundary of the model as the datum edge. Edge 1, Edge 2, Edge 3 and Edge 4 represent the positions which have a distance of 1 times, 5 times, 10 times, 15 times d1 from the datum edge. It is observed that the maximum tensile shear stress is at the interface in the film edge in the case of substrate and the maximum compressive shear stress is at the interface in the film deposited on substrate in Figure 7. The compressive shear stress and the tensile shear stress show a decreasing trend upon moving away from the film edge. The small value of tensile shear stress at the top surface of the film is due to the free surface phenomenon. The shear stress value can determine an adhesive strength of the coatings, as they are equivalent to each other [9].

**Figure 8:**
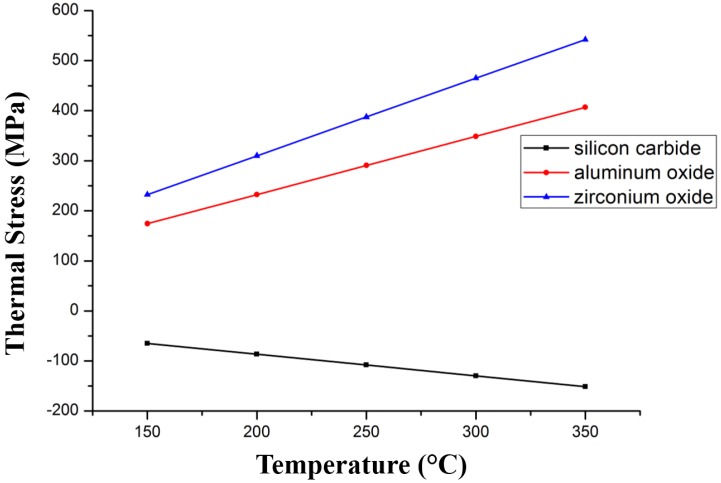
Thermal stress variation with temperature and the material of substrate.

Figure 8 shows the effect of different ceramic materials on thermal stress. We chose three types of high temperature resistant ceramic (silicon carbide ceramic, aluminum oxide ceramic, and zirconium oxide ceramic) as the substrate and W-3Re as the film. We also choose d_2_ = 100 μm, d_1_ = 1 μm. It is observed that whether tensile stress or compressive stress is generated in which kind of ceramic substrate, the thermal stress increases as the deposition temperature increases, and silicon carbide has good performance, and for this reason is chosen as the substrate material.

**Figure 9:**
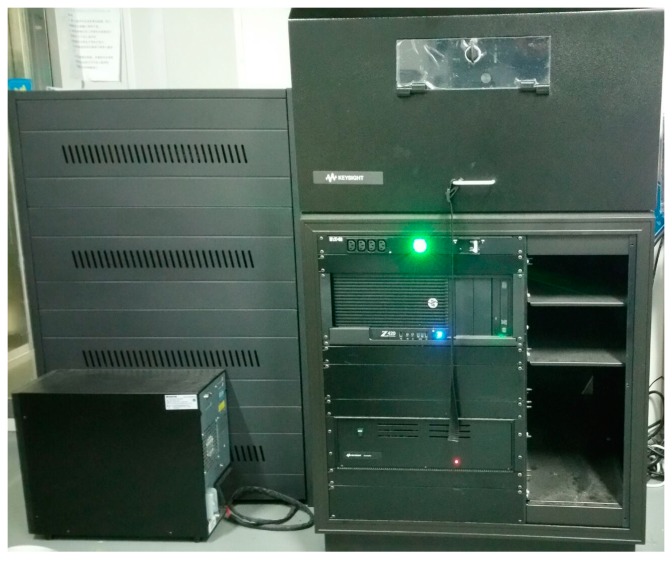
Nano-indentation film test system.

To compare with the simulation results of FEA, nano-indentation experiments are done to measure and calculate the film stress, shown in Figure 9. A nano-indentation apparatus (G200, Agilent Technologies Inc. (China), Beijing, China) with a three sided pyramid Cube-Corner indenter (54.7356°) is used to test the hardness and elastic modulus of films with the selected loading process [10].

W-3Re film was applied on different ceramic materials (silicon carbide ceramic, aluminum oxide ceramic, and zirconium oxide ceramic), and the chose d_2_ = 100 μm, d_1_ = 1 μm, T = 100 °C as the parameters of the W-3Re films. Load on the given indentation depth is 2000 nm with a speed of 0.064 nm/s. The results, such as elastic modulus, hardness, loading curve, are measured. We chose the model derived by Suresh and ABAQUS to calculate the film stress [11]. The results are shown in Table 3. It can be observed that the measurement results are in accordance with the simulation model.

Considering a film under high temperature thermal stress mismatch will cause damage, an orthogonal range analysis is done to find out the main factors influencing the thermal stress of the W-3Re film of TFTCs, so as to determine the best factor level for the thermal combination effect. As the substrate material has been selected, W-3Re film thermal stress is mainly affected by the thickness of the ceramic substrate, the expansion coefficient of tungsten-rhenium film thickness, and the temperature. According to the three factors and three levels of orthogonal experimental requirements, each factor of the three different levels is determined in Table 4. Using the FEA model to do the range analysis of the film thermal stress, each factor at different levels of the maximum and minimum values of the difference are found out through the three factors and three levels orthogonal experiment. The impact of the three factors on the stress and deformation of thin films is analyzed, and the optimization to reduce the film stress collocation scheme is determined. In addition, the temperature is chosen based on the operating temperature of the W-3Re film.

We select film stress as the index, the results are shown in Table 5. Table 6 shows the results of the range analysis of thermal stress in W-3Re films.

K_1_, K_2_, K_3_ show the sum of film stress at the levels 1, 2, 3 respectively, k_1_, k_2_, k_3_ show the average values of film stress at the level of 1, 2, 3 respectively, R is the range value. The thermal stress data from Table 5 shows that the W-3Re films stress reaches a minimum value of 259.2 MPa in experiment 1. The substrate thickness is 100 μm, the W-3Re film thickness is 1 μm, and the temperature is 600 °C. According to the calculated thermal stress range results, the primary and secondary factors affecting the numerical value are determined. The thermal stress range analysis in W-3Re films shows that in our design of the size and working conditions, the temperature has a greater influence on the thermal stress than the film thickness, and the influence of film thickness on thermal stress is the lowest, relatively. The situation is similar due to the similarity of the W-3Re and W-25Re materials.

## 4. Experiments and Results

### 4.1. TFTC Preparation Experiments

Based on range analysis, we choose the dimension parameter used in experiment 1 for the optimization scheme of TFTCs to confirm the results of the simulation analysis. Suitable tungsten-rhenium TFTC structures were prepared and tested.

**Figure 10:**
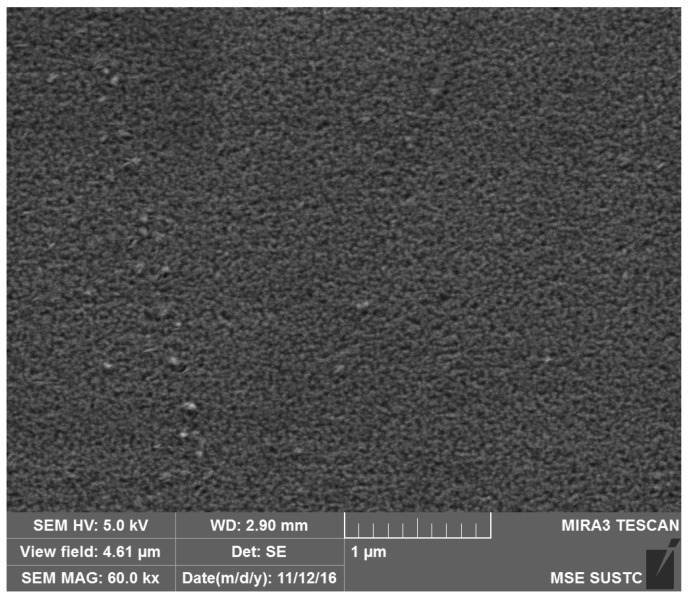
The SEM result of W-3Re TFTCs.

We choose W-3Re film for the composition and morphology of the films. The scanning electron microscope (SEM, type: Tescan Mira 3, Shanghai, China) results are shown in Figure 10. The test parameters are also shown in the figure (HV: 5 kV, WD: 2.9 nm, MAG: 60 kx). It is observed that the films exhibit dense structure.

**Figure 11:**
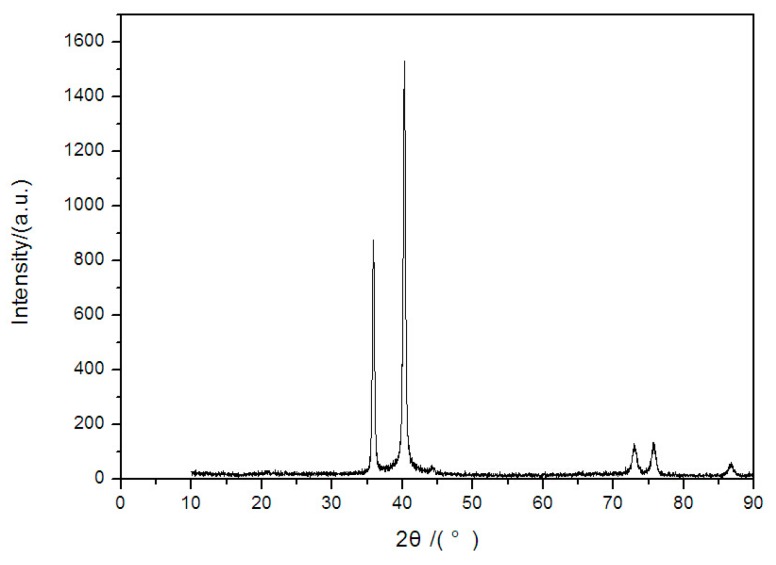
The XRD result of W-3Re TFTCs.

**Figure 12:**
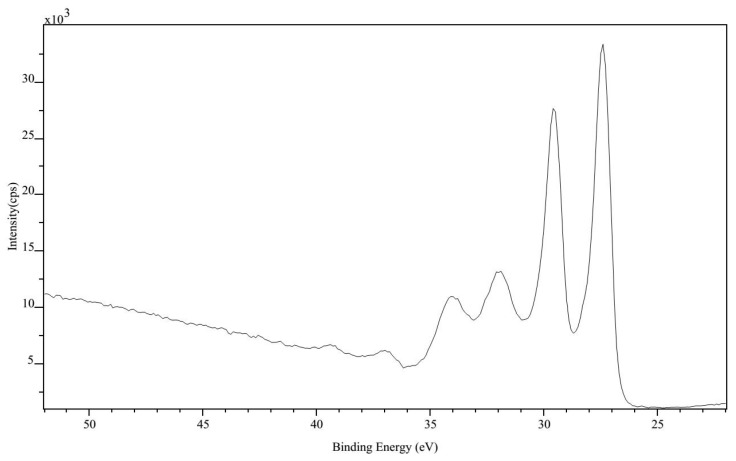
The XPS result of W-3Re TFTCs.

The X-ray diffraction (XRD, type: d/max-2400, Rigaku Corporation (China), Beijing, China) and X-ray photoelectron spectroscopy (XPS, type: Axis Ultrabld, SHIMADAZU (China), Shanghai, China) results are shown in Figure 11 and Figure 12 respectively, which show the composition elements and atomic ratio of W-3Re film to determine the material composition accurately after preparation. It is observed that the TFTC preparation was successful.

The TFTC sensor materials used include tungsten-rhenium, silicon carbide ceramics, and mixed high temperature binder. Ceramic substrate is made by powder sintering. It’s cut into the proper size and polished to make films sputter on the surface successfully. The substrate is ultrasonically cleaned with deionized water, acetone, etc. TFTCs are fabricated by a micromachining process using sputter technology and UV lithography. The TFTCs are patterned with stenciled shadow masks. The TFTCs (tungsten-3% rhenium versus tungsten-25% rhenium) are deposited directly onto the substrate surface. Some related process parameters are listed in Table 7.

**Figure 13:**
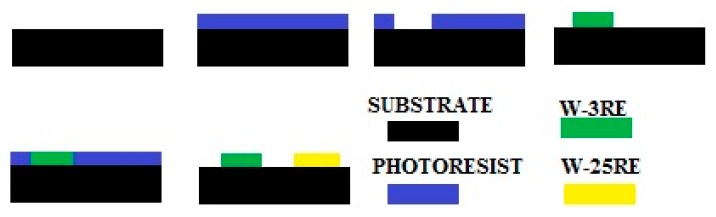
Brief schematic of TFTCs.

A diagram of the process is shown in Figure 13.

Then TFTCs sensors are heat treated in a protective atmosphere. Finally, the cold terminal films are connected with wires using a mixed high temperature binder at 120 °C.

### 4.2. Thermal Response Experiments of TFTCs

**Figure 14:**
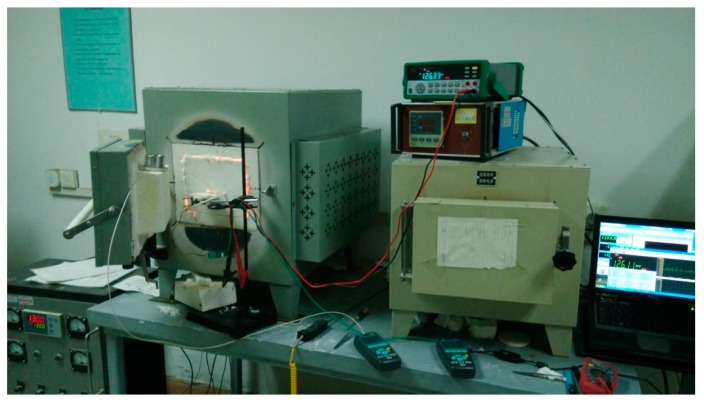
The static thermal experiment environment.

A static thermal environment was used for testing the performance of TFTCs. A muffle furnace (KSY-12-16S, Shanghai Shi Yan Electric Furnace Corporation, Shanghai, China) with a maximum high temperature of 1800 K is used to provide the test environment shown in Figure 14. An OMEGA B type thermocouple device, which has a measurement range from room temperature to 1800 K, is adopted for calibration. The output signal was recorded by an AZ8856 temperature recorder (precision 0.1 K, AZ Instrument, Taichung, Taiwan) and a K2000 type voltage tester (precision 1 μV, TEKTRONIX (China), Shanghai, China).

**Figure 15:**
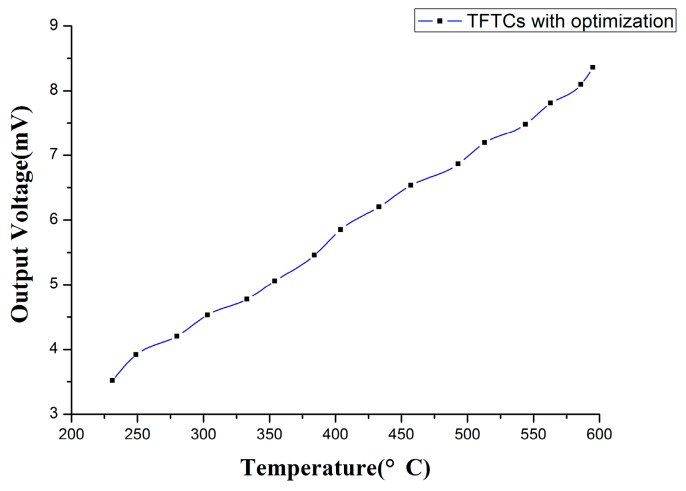
The output of TFTCs with optimization.

The experiment result is shown in Figure 15. The maximum thermoelectric voltage of TFTCs subjected to a temperature difference of 600 °C was 8.46 mV on SiC substrates. Optimized TFTCs could resist for an hour when the temperature is kept at 600 °C.

**Figure 16:**
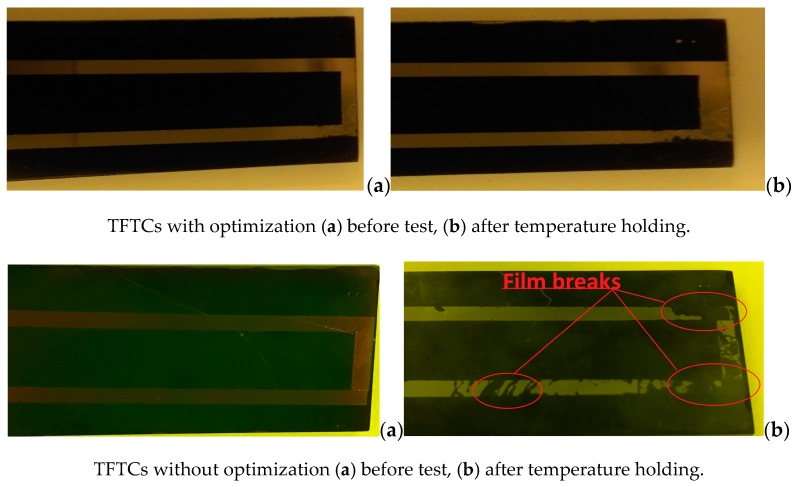
The fabricated TFTCs.

TFTCs without dimensional parameter optimization were tested for comparison. These can only bear the temperature for one minute before the film rupture even when the material is the same. The TFTCs without optimization fall off and break. The substrate thickness of TFTCs without optimization is 3 mm, the W-3Re films thickness 50 μm, the length of the film and substrate is 8 cm. Two types of W-3Re TFTCs are prepared and tested when a similar temperature difference is applied, as shown in Figure 16. It can be observed that the film is intact before the test and the films of TFTCs without optimization fall off and break after holding at the test temperature. TFTCs without optimization seriously fall off and break at the hot junction, while TFTCs with optimization are nearly intact after temperature holding. There were no significant changes in the components of the TFTCs. The simulation shows the thermal stress in TFTCs with optimization is 259.2 MPa and 401.8 MPa in TFTCs without optimization at 600 °C, while the thermal stress in ceramic substrate is relatively minutes. In addition, the XPS result of TFTCs without optimization after temperature holding shows the material composition of undamaged film remains stable. It is observed that the TFTCs without optimization fall off and break after exposure to a constant high temperature at 600 °C due to their coefficient of thermal expansion mismatch.

## 5. Conclusions

Range analysis of thermal stress of tungsten-rhenium thin film thermocouples based on ceramic substrates is presented to explain the falling off and breaking problem caused by the mismatch of the thermal stress in TFTCs and substrates. FEA and numerical analysis is used to calculate the thermal stress, and nano-indentation experiments are done to measure and calculate the film stress to compare with the simulation results. The relationship between substrate materials and dimensional parameters is analyzed and the optimal design of tungsten-rhenium TFTCs is reported. The range analysis of thermal stress in W-3Re films shows the temperature has a greater influence on the thermal stress than the thickness of the film, and the influence of film thickness on thermal stress is relatively the lowest.

Static high temperature tests are carried out after the TFTCs are fabricated. The maximum thermoelectric voltage of TFTCs subjected to a temperature difference of 600 °C was 8.46 mV on SiC substrates. It is observed that the TFTCs without optimization fall off and break after exposure to a constant high temperature of 600 °C due to the coefficient of thermal expansion mismatch, and TFTCs with optimization could bear a temperature of 600 °C for an hour. The result shows the optimization design based on the thermal stress range analysis can effectively reduce the damage caused by the mismatch of the thermal stress in rhenium thin film thermocouples based on ceramic substrates in a certain temperature range.

## Figures and Tables

**Table 1:** Table of material parameters [6].

Materials	Poisson’s Ratio	Young’s Modulus (GPa)	Coefficient of Thermal Expansion (10^−6^/K)
silicon carbide ceramic	0.17	360	3.67
aluminum oxide ceramic	0.29	390	7.7
zirconium oxide ceramic	0.22	170	10
tungsten-3% rhenium	0.28	400	4.57
tungsten-25% rhenium	0.28	363	5.05

**Table 2:** Definitions of the symbols in Equation (1).

Symbol	Definition
$E_{ef}=E_{f}/(1-v_{f})$	Effective Young’s modulus of the coating
$E_{es}=E_{s}/(1-v_{s})$	Effective Young’s modulus of the substrate
*ν_f_*	Poisson’s ratio of the coating
*ν_s_*	Poisson’s ratio of the substrate
*h*	Coating thickness
*H*	Substrate thickness
*T_D_*	Deposition temperature
*T_r_*	Room temperature
*α_f_*	Thermal expansion of coefficients of the coating
*α_s_*	Thermal expansion of coefficients of the substrate

**Table 3:** Comparison of simulation result and nano-indentation results (unit: MPa).

Material of Substrate	FEA Results	Analytical Results	Nano-Indentation Results
silicon carbide	43.4	43.3	47.0
aluminum oxide	115.9	116.4	121.2
zirconium oxide	153.9	155.1	158.8

**Table 4:** Allocation table of orthogonal test influence factors.

Level	Substrate Thickness/μm	W-3Re Films Thickness/μm	Temperature/°C
1	100	1	600
2	500	2	700
3	1000	3	800

**Table 5:** Experimental scheme and results of three factors and three levels.

Experiment	Substrate Thickness/μm	W-3Re Films Thickness/μm	Temperature/°C	W-3Re Films Stress/MPa
1	1	1	1	259.2
2	1	2	2	290.8
3	1	3	3	320.1
4	2	1	2	312.4
5	2	2	3	354.1
6	2	3	1	263.4
7	3	1	3	358.1
8	3	2	1	267.7
9	3	3	2	311.1

**Table 6:** Rang analysis of thermal stress in W-3Re films (unit: MPa).

Factors	Substrate Thickness	W-3Re Films Thickness	Temperature
K_1_	870.1	929.7	790.3
K_2_	929.9	912.6	914.3
K_3_	936.9	894.6	1032.3
k_1_	290	309.9	263.4
k_2_	310	304.2	304.8
k_3_	312.3	298.2	344.1
R	22.3	11.7	80.7

**Table 7:** Allocation table of orthogonal test influence factors.

Process Parameters	Sputtering Pressure	Vacuum Degree	Sputtering Time	Sputtering Power	Argon Flow Rate
Numerical Value	1.0 pa	10^−^^5^ Pa	2 h	150 W	60 sccm
